# The Role of Conformational Dynamics in the Recognition and Regulation of Ubiquitination

**DOI:** 10.3390/molecules25245933

**Published:** 2020-12-15

**Authors:** Domarin Khago, Ian J. Fucci, Robert Andrew Byrd

**Affiliations:** Structural Biophysics Laboratory, Center for Cancer Research, National Cancer Institute, P.O. Box B, Building 538, Frederick, MD 21702-1201, USA; domarin.khago@nih.gov (D.K.); ian.fucci@nih.gov (I.J.F.)

**Keywords:** ubiquitin, ubiquitination, E2, really interesting new gene (RING) E3, NMR, conformational dynamics, relaxation, allostery

## Abstract

The ubiquitination pathway is central to many cell signaling and regulatory events. One of the intriguing aspects of the pathway is the combinatorial sophistication of substrate recognition and ubiquitin chain building determinations. The abundant structural and biological data portray several characteristic protein folds among E2 and E3 proteins, and the understanding of the combinatorial complexity that enables interaction with much of the human proteome is a major goal to developing targeted and selective manipulation of the pathway. With the commonality of some folds, there are likely other aspects that can provide differentiation and recognition. These aspects involve allosteric effects and conformational dynamics that can direct recognition and chain building processes. In this review, we will describe the current state of the knowledge for conformational dynamics across a wide timescale, address the limitations of present approaches, and illustrate the potential to make new advances in connecting dynamics with ubiquitination regulation.

## 1. Introduction

**Figure 1:**
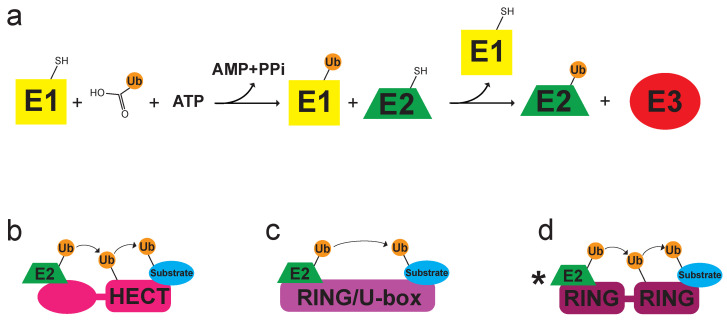
(**a**) Schematic representation of the ubiquitination pathway involving E1-E2-E3 enzymes through a concerted, ATP-driven process. There are two major classes of E3 ligases that dictate ubiquitin transfer: (**b**) homologous to E6AP C-terminal (HECT) domain, where a conserved, catalytic cysteine becomes charged with ubiquitin before the final transfer to substrate; and (**c**) really interesting new gene (RING)/U-box domain, where the stabilized E2∼Ub conjugate directly transfers ubiquitin from E2 to substrate. There is an additional small class of proteins called (**d**) RING-in-between-RING (RBR) that behave as a RING/HECT hybrid, where one RING domain recruits an E2 which then transfers ubiquitin to the second RING-like domain.

Ubiquitination is a powerful post-translational mechanism involved in myriad cellular signaling and proteasomal degradation pathways. Specific ubiquitination depends on the sequential cascade of substrate–ubiquitin (Ub) chain building through an ATP-driven, concerted process among three classes of enzymes (Figure 1a): ubiquitin-activating enzymes (E1), ubiquitin-conjugating enzymes (E2), and ubiquitin ligating enzymes (E3) [1,2,3,4,5]. The human genome encodes for ∼2 E1s, ∼40 E2s, and >600 E3s, where collectively, these enzymes control the ubiquitination of the entire cellular proteome [5,6,7,8]. Substrates can be covalently modified through a single ubiquitin (monoubiquitination), multiple ubiquitins (multimonoubiquitination), or a chain of ubiquitins (polyubiquitination) [3,6,7]. The specificity of ubiquitination relies on the E2:E3 complex pairing, in particular, the E3 class-type, which designates ubiquitin transfer occurring from either E2 or E3 towards substrate as well as linkage specificity [6,9].

E3 ligases are primarily made up of two classes in eukaryotes: homologous to E6AP C-terminal (HECT) and RING/U-box ligases (Figure 1b,c, respectively) [3,4,6,9,10]. HECT E3 ligases contain a globular domain with a conserved, catalytic cysteine that becomes charged with ubiquitin that is passed from an E2∼Ub (where ∼ represents a thioester bond) before the final transfer to substrate [9,11]. Conversely, RING/U-box ligases, which make up the largest class of E3s, stabilize the E2∼Ub conjugate in preparation for the direct transfer of ubiquitin from E2 to substrate [3,6,10]. The stabilization of the E2∼Ub thioester conjugate is achieved through the interaction of the RING/U-box domain with E2 [5]. There is an additional small class of proteins called RING-in-between-RING (RBR) that behave as a RING/HECT hybrid (Figure 1d) [3,12,13]. One RING domain recruits an E2 which then transfers ubiquitin to the second RING-like domain [3,12]. For the purpose of this review, we will be focusing on the RING E3 ligases.

RING and RING-fingerlike E3 ligases form the large majority (>500) of E3s in humans [14]. Almost half of RING E3 ligases contain a conserved arginine that interacts with both the E2 and ubiquitin tail (e.g., the conserved Arg400 in Hrd1), causing a conformational change where the E2∼Ub conjugate becomes restrained and prepared for nucleophilic attack [5,15]. RING E3s bind to conserved portions of E2s in order to regulate activity. Certain RING E3s contain ancillary regions that bind the “backside” region of E2s to further enhance the affinity of the E2∼Ub:E3 complex [5]. Three-dimensional structures of RING E3s show a hydrophobic core “finger” domain of conserved, cross-braced cysteine and histidine residues that coordinate two zinc atoms [4,5,6], while some RING E3s have cysteines swapped with other residues that coordinate zinc [6]. E2∼Ub:RING surface contacts have been of continued interest in order to understand how RING binding affects the overall mechanism of ubiquitin transfer to substrate. Undoubtedly, there exists a subset of residues in E2, as well as their precise side-chain packing, which control the allosteric communication between E2 and E3 ligase active sites [16].

**Figure 2:**
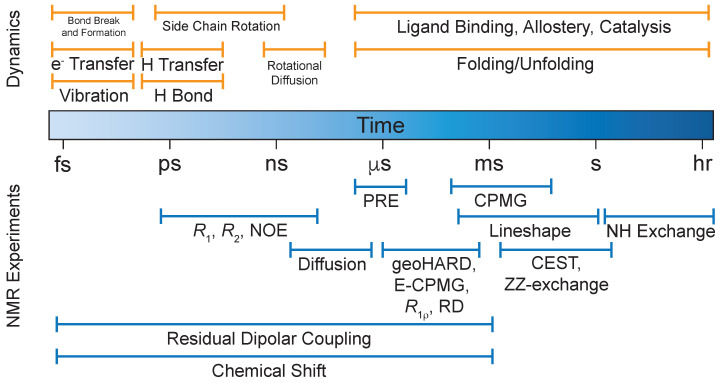
Accessible timescale of NMR experiments. The top portion portrays the protein dynamics and the bottom portion corresponds to the NMR methods, including geoHARD and E-CPMG, used to probe those events.

Allosteric events, such as those observed in E2:E3 pairs, are often considered primarily as shifts in structure or conformations that promote interactions. However, these events may be dynamic in nature and revealed as minor, high energy states that become more populated as a result of binding one domain, and lead to significant enhancement of binding another domain to form the complete complex. In such cases, the structural shifts may be less dramatic, while the energetic outcome is significant. The relative equivalence of E2 structures raises the question of whether there exists an energy landscape of minor populated species that become favorable as the complex is formed. Exploration of such landscapes is difficult using classical structural techniques; however, solution-state nuclear magnetic resonance (NMR) spectroscopy is one of several techniques that can explore protein structure and dynamics through correlation and relaxation experiments. NMR relaxation studies can provide detailed information on the time-dependent fluctuations of proteins at atomic resolution [17]. These fluctuations include internal motions characterizing distinct protein conformations of domains or subunits on a wide range of timescales representative of a myriad of functional implications [17,18,19,20]. Conventional measurements of backbone ^15^N amide dynamics entails the longitudinal relaxation rate (R${}_{1}$), the transverse relaxation rate (R${}_{2}$), and the heteronuclear Overhauser effect (hetNOE) [19,21]. These relaxation rates represent motional frequencies on the picosecond-to-nanosecond (ps-to-ns) timescale (Figure 2) and are analyzed to quantify key parameters: rigidity of the amide bond vector (S^2^), intramolecular correlation times (τ${}_{e}$), and the overall molecular tumbling time (τ${}_{c}$) [19,20,21]. Traditionally, the timescales more aligned with binding, allostery, and catalysis events are investigated using Carr–Purcell–Meiboom–Gill (CPMG) and R${}_{1\rho }$ relaxation dispersion (RD) methods, which provide a detailed insight to kinetic (k${}_{\mathrm{ex}}$), thermodynamic (p${}_{a}$, p${}_{b}$), and structural information (Δω, Δδ) in the microsecond-to-millisecond (μs-to-ms) timescale (Figure 2) [17,22,23]. These methods have a finite timescale, associated with instrumental limitations, that allows observations of dynamic processes in the hundreds of μs to 10 ms. There are now new methods emerging that will extend this range down to the range of 5–10 μs [24,25,26]. Finally, the slower time scale of 10–200 ms is covered by ZZ-exchange and chemical exchange saturation transfer (CEST) methods [27,28]. The extracted NMR relaxation parameters can be coupled with molecular dynamics (MD) simulations to provide a structural model. Recent advancements in hardware and enhanced sampling techniques have allowed for MD simulations to capture dynamic events on the μs to ms timescale, providing complementary information to the NMR methods presented here [29]. Through the judicious choice of current and emerging solution NMR methods, it is becoming possible to carefully examine the dynamic energy landscape of E2, E3, and E2∼Ub systems, as well as their complexes, to decipher the allosteric regulation of the ubiquitination pathway. This review will consider the present state of applications to the E2 and E3 proteins and complexes, summarize the insights obtained as well as the limitations, and postulate where new tools may expand insight into the pathway.

**Figure 3:**
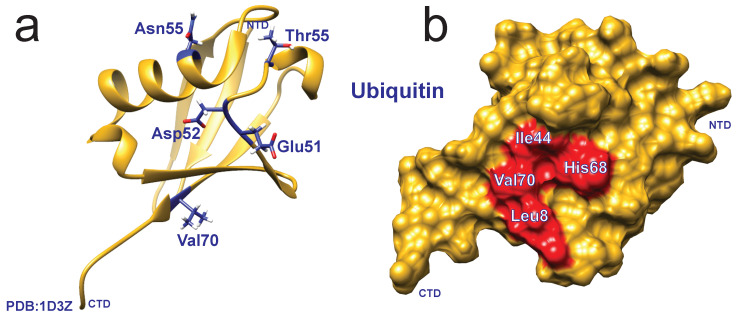
Structural representations of ubiquitin (PDB: 1D3Z). (**a**) Ribbon representation of ubiquitin, where residues showing conformational exchange are highlighted in blue shown through NMR relaxation experiments [30,31,32,33,34,35,36,39,40]. (**b**) Surface representation of ubiquitin, where residues in red portray the hydrophobic patch.

Often regarded as a simple post-translational modification, the conformational dynamics of ubiquitin itself can play an essential role in cell signaling and degradation mechanisms, making it an important case to understand for functional significance. Multiple backbone relaxation studies observed chemical exchange broadening for residues Ile23 and Asn25 (N-terminus of the α-helix) [30,31,32,33,34,35]. Conventional relaxation experiments were unable to probe slower motional modes (μs to ms), which led to the development of measuring backbone dynamics in supercooled water [36]. These results were later confirmed with CPMG and R${}_{1\rho }$ RD experiments [37,38,39,40]. These experiments identified additional conformational exchange in Val70 (strand β${}_{5}$, forms hydrophobic patch with Leu8 and Ile44) [36], Glu51-Asp52 (loop connecting strands β${}_{4}$ and β${}_{5}$, a component of the hydrophobic patch with Leu8 and Ile44) [39], and Thr55 (loop between strand β${}_{4}$ and short second 3${}_{10}$ helix) (Figure 3) [40]. Interestingly, conformational dynamics within free ubiquitin have been shown to play a role in selectivity and recognition by deubiquitinating-enzymes (DUBs). Through a series of mutagenesis and relaxation studies, ubiquitin mutants were identified that bias the conformational dynamics such that one subclass of deubiquitinating enzymes bind with higher affinity than others [41]. In a separate study exploring the regulation of ubiquitin through phosphorylation, dynamics at multiple timescales were probed using a series of NMR methods. CEST experiments were used to quantitate the population of the minor state of ubiquitin in which a buried serine becomes exposed, previously shown to be responsible for phosphorylation by PINK1 [42]. The important minor state, which shows exchange on the millisecond time scale (Figure 2), was proposed to be the state that binds to PINK1 and promotes phosphorylation [42]. The bias to certain conformational states suggests modulation of the population of states as a function of the environment and binding partners. Importantly, in the absence of binding partners, the studies on ubiquitin provide a baseline understanding to the intrinsic motions of the molecule.

A wealth of structural data exists describing the protein–protein interactions within the ubiquitination pathway. These structures can be described as “snapshots”, but they do not describe the various conformational states and their interconversions, which could elucidate a more detailed mechanism of interaction. The dynamics of ubiquitin and the studies described herein provide a window into the allostery and recognition within the ubiquitination pathway. Exploring techniques to tease out conformational dynamics of these proteins and complexes may provide a more complete understanding of the ubiquitination pathway.

## 2. Intrinsic Dynamics within E2s

**Figure 4:**
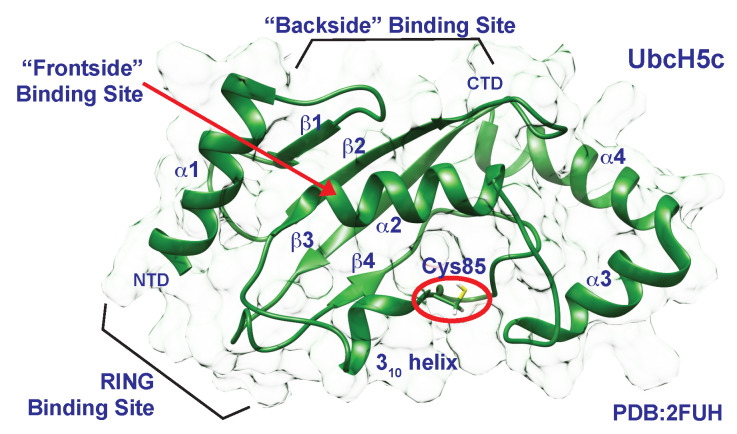
Structural representation of UbcH5c (PDB 2FUH) portraying the conserved E2 Ubc fold. The fold consists of 5 external α-helices, a backside 4-stranded antiparallel β-sheet, and a short 3${}_{10}$ helix proceeding the catalytic cysteine. The catalytic cysteine is indicated with a red oval.

First described as intermediate “carriers” of ubiquitin, E2s bind to E3s to catalyze the transfer of ubiquitin from E2 to substrate (Figure 1) [1,43]. These proteins are, at their smallest, merely twice the size of ubiquitin, yet facilitate interactions with cognate E1s and E3s, determine thiol or amine reactivity, and dictate chain specificity [3,12,44]. E2s share a conserved Ubc fold consisting of 5 α-helices with one crossing over the middle of the protein (termed the “frontside”), a backside 4-stranded antiparallel β-sheet, and a short 3${}_{10}$ helix preceding the catalytic cysteine (Figure 4).

A structural genomics screen of human E2s showed that the shared Ubc core is remarkably similar, with the most conserved portions located in the active site and overlapping E1/E3 interaction region (α${}_{1}$ and β${}_{3}$β${}_{4}$ loop) [45]. Surface residues are less conserved, potentially providing unique interaction surfaces with their cognate E3s. These regions have been previously identified and described by van Wijk and Timmers in their “shell model” [46]. Conserved regions in this model are grouped as first shell residues, those that are required for activity and loading by E1, with subsequent shells composed of less conserved residues. Integral to this model is the transfer of information from third shell residues (surface-exposed backside and E3-interacting residues) to first shell residues through the critical second shell residues [46]. A combined mutational and statistical coupling analysis of conserved residues in UbcH5b identified an allosteric network of these so-called second shell residues upon RING binding to E2 [16]. The Ubc fold is capable of transducing a wide array of information from the surface residues inward to the catalytic center. Further, we suggest this transduction of information may correlate to changes in the conformational dynamics of these critical residues. The following section features the limited examples that highlight the conformational dynamics of E2 enzymes and their role in regulation and catalysis.

Understanding the intrinsic dynamics of uncharged E2s provides a baseline by which activated states can be analyzed. In an early investigation of UbcH5b, significant conformational dynamics on the μs to ms timescale were not observed in CPMG and R${}_{1\rho }$ RD experiments (Figure 2). To date, minor (invisible) states in E2s have not been revealed to be in slow exchange by tools such as CEST. However, the calculated NMR structure shows that a conserved asparagine, purported to stabilize the oxyanion intermediate of the Ub-substrate transition state [9], undergoes conformational exchange that may be relevant to catalysis [47]. One caveat of this study is that it was performed at 27 °C, where the motions may be outside of the timescale of these experiments. More recent studies of uncharged Ube2g2 and UbcH5b using Carr–Purcell–Meiboom–Gill relaxation dispersion (CPMG-RD) at reduced temperatures in addition to heteronuclear adiabatic relaxation dispersion (HARD) experiments identified dynamics in the μs to ms timescale on the backside of both molecules [23,26,48]. Temperature reduction slows down the dynamic processes enough to be observed by CPMG-RD while HARD experiments provide access to wider timescales (Figure 2), allowing for quantification of the same motions at higher temperatures. In the absence of its binding partners, Ube2g2 exchanges between multiple conformations. These dynamics become quenched in Ube2g2 upon addition of the G2BR (Ube2g2 binding region) of the E3 gp78, which binds the backside. This stabilization of the molecule provides a conformation that promotes RING binding, thus increasing the affinity 50-fold [49]. The increased affinity suggests that the energy landscape for Ube2g2 exhibits different conformational states, and the population of the effective RING binding states is considerably lower in the absence of G2BR.

In addition to binding E3s, oligomerization of E2s may stabilize productive states through these same allosteric sites, namely, the backside [50,51]. The formation of E2 homo-oligomers in vitro has been suggested as a possible mechanism for bringing together two or more ubiquitin molecules in close proximity and for forming ubiquitin chains [50]. Mms2:Ubc13, a well characterized system, involves the formation of the hetero-oligomer between an E2 and a ubiquitin E2 variant (UEV), which are noncatalytic E2-like proteins able to bind Ub [52,53]. The Mms2:Ubc13 complex is able to form diubiquitin through the coordination of substrate ubiquitin by Mms2 (UEV) towards the active site of Ubc13 [54]. Conversely, the active conformation of Ube2S is stabilized through its interaction with substrate ubiquitin directly [55]. Though these mechanisms are not mutually exclusive, it is interesting to speculate that there may be a difference in conformational dynamics between E2s that act as ubiquitin chain elongators and those that act in monoubiquitination or apply the first ubiquitin of the chain. There are also examples of autoubiquitination of E2s through the flexibility in the active site, exposing a conserved lysine (present in about 25% of human E2s in the 3${}_{10}$ helix), which may function as a form of self-regulation [56,57].

Backside binding of ubiquitin to E2s in vitro has been reported, indicating significant overlap in interaction surfaces for binding partners [44]. Though many cases indicate that backside binding has a positive allosteric effect, ubiquitin binding to the backside of Ube2E3 seems to prevent chain building [58]. The previous examples underscore that we are just starting to unravel the complex web of interactions between E2s, E3s, and ubiquitin as well as how these effects are transduced by changes in conformational dynamics. Hidden within the sequence and structure of every E2 is the capability of exchanging between states conducive for protein–protein recognition and catalysis. Clearly, E2s are not merely carriers of ubiquitin, but play essential roles in its activation and regulation.

## 3. E2∼Ub Conjugates: Dynamic Intermediates

After recruitment of an E2 by the E1, a transthiolation reaction charges the E2 with ubiquitin to form an E2∼Ub conjugate, the form most abundant in the cell [3,59]. Therefore, understanding the structure and conformational dynamics of the E2∼Ub conjugate is paramount to understanding the cellular function of these enzymes. Early biochemical studies showed that ubiquitin can be transferred from E2∼Ub to free lysine (as well as other small molecules containing primary amines attached to primary carbons) in an E3-independent manner [43]. The specificity of this reaction and variation in reactivity between E2s indicates that this is, in fact, a catalyzed reaction, suggesting that these small molecules can induce an active conformation. A similar assay of E2∼Ub reacted with free amino acids showed that while most E2s can transfer to both lysine and cysteine, UbcH7 is specific to cysteine and cannot transfer ubiquitin to lysine [12]. There are also E2s that transfer ubiquitin to serine or threonine residues and others capable of *N*-terminal ubiquitination [44]. Structures of E2∼Ub conjugates remain somewhat elusive as the instability of this intermediate makes it particularly challenging to study.

Pioneering work by Miura et al. provided the first, structural study of ubiquitin conjugated to an E2 [60]. The instability of the thioester between the catalytic cysteine and ubiquitin C-terminus was overcome using an active site Cys→Ser mutant, replacing it with the considerably more stable (oxy)ester. The Cys→Ser mutant—also a Cys→Lys mutant that forms an isopeptide (amide) bond with ubiquitin—has been widely used to generate stable E2-Ub (n.b., the hyphen (-) represents a covalent bond other than a thioester), which are more amenable to techniques such as X-ray crystallography or NMR spectroscopy [55,61,62,63,64]. These early structural studies identified that the interaction between the covalently-bound ubiquitin and E2 surface is largely concentrated in the C-terminal tail of ubiquitin and in a cleft near the active site cysteine of E2. Additionally, ubiquitin residues Lys48 and Gln49 were found to interact with the Ube2A surface [60,65,66]. In addition to production of stable ester- or amide-bonded ubiquitin, a number of groups have opted for in situ formation of E2∼Ub in conjunction with NMR spectroscopy in an effort to map the interaction surfaces between various E2s and ubiquitin [65,66,67]. These studies identified a binding cleft on E2 intended for the ubiquitin C-terminal tail proximal to the catalytic cysteine, as well as a distal patch shown to interact with the hydrophobic patch of ubiquitin (Ile44) (Figure 3b) [67]. It should be noted that a biotinylated ubiquitin C-terminal peptide (LRLRGG) can undergo transfer through the E1-E2-E3 cascade to produce a biotinylated substrate with a significantly reduced affinity for E2 [68,69]. While the ubiquitin C-terminus is sufficient to drive catalysis, these ancillary interactions between the ubiquitin surface, particularly the hydrophobic patch, greatly increase the affinity of the ubiquitin. The surface interactions between ubiquitin and E2 suggest that allostery plays an important role and may provide a mechanism for activation and lysine specificity.

**Figure 5:**
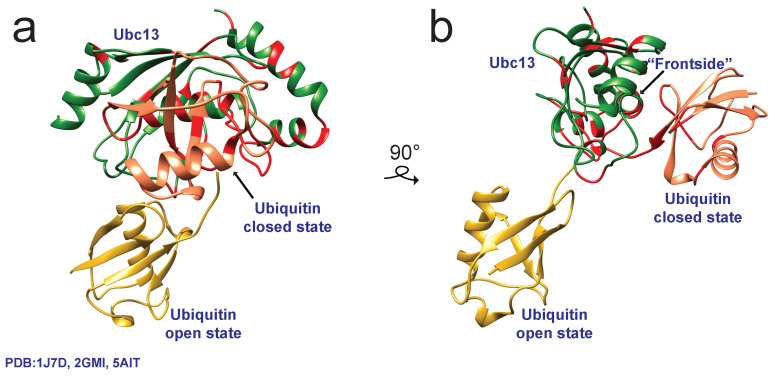
(**a**) Composite structural representation of Ubc13 (green, PDB 1J7D) with ubiquitin in either the open state (gold, PDB 2GMI) or the closed state (coral, PDB 5AIT). (**b**) Ubc13-Ub complex rotated 90° to the right showcasing the swing from the open state to the closed state. The open state representation was modeled by superimposing human Ubc13 onto the crystal structure of yeast Mms2:Ubc13-Ub where only ubiquitin is visible. In the closed state structure, RNF4-RING and Mms2 are omitted for clarity. Residues mapped in red for both structures represent significant chemical shift perturbations (CSPs), indicating the binding surface between Ubc13 and ubiquitin. These protein–protein interactions were confirmed through SAXS and paramagnetic relaxation enhancement (PRE) in addition to CSP experiments and have been shown to be relevant to the formation of the active state [70].

The interactions between the ubiquitin and the E2 surface would later be revealed to be relevant to ubiquitin transfer. The NMR relaxation study by Pruneda et al. showed that the ubiquitin moieties of ester-linked UbcH5c-Ub and Ubc13-Ub are quite dynamic [70] and transition between two-states, referred to as closed and open states, where the ubiquitin moiety is or is not interacting with the E2 frontside, respectively (Figure 5). Upon interaction with a cognate U-box E3, E4BU, the ubiquitin of UbcH5c-Ub is biased toward the closed conformation, while maintaining a fair amount of flexibility [10,71]. A recent study uses single molecule Förster Resonance Energy Transfer (FRET) to examine the mobility of ubiquitin in the isopeptide-linked Mms2:Ubc13-Ub. The authors reported free Ubc13-Ub accesses the widest number of FRET states, as expected for a dynamic ubiquitin moiety. Unexpectedly, upon binding to the UEV Mms2, the low FRET open conformation becomes more populated, reflecting a previously reported so-called stochastic gating mechanism for Ubc13 in which access to the active site by the ubiquitin C-terminal tail is regulated by ps-to-ns motions in the E2 [72]. Time-resolved FRET in the presence of the RNF4 RING domain reveals that Mms2:Ubc13-Ub:RNF4 adopts the closed conformation during catalysis, providing real-time evidence of the conformation of bound ubiquitin during transfer [73]. A snapshot of the active conformation is shown by previous crystal structures of Mms2:Ubc13-Ub:RNF4 in complex with a second ubiquitin molecule, where Lys63 is poised for nucleophilic attack of the thioester [74].

The works presented here provide the evolution of our understanding of the structure and dynamics of E2∼Ub conjugates. Difficulties in forming stable E2∼Ub conjugates have hampered progress in this area, and much of our understanding comes from the well-behaved Ubc13 and UbcH5 family of E2s. The conformational dynamics of E2s within E2∼Ub conjugates that contain unique structural features, such as C- and *N*-terminal extensions or insertions, remain largely unexplored, preventing meaningful analyses of common mechanisms among this diverse class of enzymes. The closed conformation of ubiquitin within the conjugate is regarded as catalytically active; however, the existence of low-population excited states within this closed conformation has not been thoroughly investigated. Quantifying the conformational dynamics of the conjugate alone provides baseline populations for the various states these two molecules adopt. The populations can then be interrogated within the broader context of the E2∼Ub:E3 ternary complex, granting insight into allosteric activation by E3.

## 4. E2∼Ub Conjugates Are Allosterically Enhanced through E3 Interactions

The combinatorial effects of ubiquitin transfer are a direct result of the concerted mechanism characterized through the E2:E3 complex that dictates cellular functions such as protein and lysosomal degradation, recruitment or impairment of binding partners, and protein localization [3]. One of many examples is the quality control of misfolded, unassembled, and regulated proteins within the endoplasmic reticulum (ER), known as the endoplasmic-reticulum-associated degradation (ERAD) pathway [14]. While approximately 70 human diseases are associated with ERAD [75], the pathway to engineer, modulate, or inhibit for a therapeutic benefit remains unclear [76]. The similarity of structure within the E2 and RING-E3 families suggests that the allosteric events involved in E2:E3 complexes is more sophisticated than lock-and-key recognition, rather, there may be regulated interconversion of minor and major states that facilitates the allostery. By elucidating the allosteric events driving formation of the E2:E3 complex, we may better understand the ubiquitination machinery and create opportunities for selectivity or modulation and hijacking the machinery for therapeutic benefits [77,78].

Several studies have examined the conformational and domain dynamics within E2:E3 and E2∼Ub:E3 systems. First, conformational biasing of the ubiquitin conjugate towards the active closed state has been observed for both UbcH5c-Ub:E4BU and UbcH5c-Ub:BRCA/BARD1, suggesting that this is a common form of activation by RING/U-box E3 enzymes. The contact surfaces were confirmed via chemical shift perturbation (CSP) measurements, paramagnetic relaxation enhancements (PREs), and mutational studies monitored by autoubiquitination assays. Other RING/U-box E3 ligases displayed a decrease in ubiquitination. CSP measurements incorporating these mutations showed the inability of E3 to shift the conjugate ensemble toward closed conformations, essentially blocking E3 enhancement of ubiquitin transfer [10]. Spotlighting the UbcH5c-Ub:E4BU ternary complex, ^15^N NMR relaxation measurements were performed to understand if closed conformations of the conjugate were in response to the change in dynamics within E2 upon E4BU binding. Diffusion tensors describing the overall tumbling confirmed that the motion of conjugated ubiquitin significantly decreases upon E3 binding [10]. Global correlation times (τ${}_{C}$) for UbcH5c within the free UbcH5c-Ub conjugate (17.7 ns) increased upon formation of the UbcH5c-Ub:E4BU ternary complex (22 ns). This increase in correlation times is consistent with the formation of the larger ternary complex [71]. Due to the flexibility of the ubiquitin moiety within the UbcH5c-Ub conjugate, quantitating global motions proved to be a challenge since interdomain motions and global motion are on the same ns timescale. These challenges can be met with the refinement of techniques applicable to larger complexes [79,80]. A mutant of E4BU (E4BU${}_{R1143A}$) formed the ternary complex with the same binding affinity as WT E4BU. Additionally, E4BU${}_{R1143A}$ in the ternary complex also reported the same ps-to-ns dynamics as WT E4BU in the ternary complex without undergoing catalysis, making the mutant a reasonable model for relaxation studies [71]. Ultimately, E3 binding to E2∼Ub conjugates promote the subsequent transfer of ubiquitin which occurs as a result of biasing towards the closed conformational state. All of these experiments measure dynamics on the ps-to-ns timescale (Figure 2), whereas measurements in the μs-to-ms timescale may reveal additional motions relevant to catalysis which have yet to be explored.

Secondly, E3 dynamics has been explored using NMR in the E3 Arkadia RING. Dynamics (^15^N backbone) experiments revealed a monomeric protein containing a rigid core with two mobile N- and C-termini. Long-range NOEs revealed a weakly populated β${}_{1}$-strand within the βββα core, separating the flexibly disordered N-terminus from the rigid core [81]. To further understand its recruitment of E2, a variety of structural and ^15^N-relaxation NMR experiments were performed on mutants of Arkadia RING, targeting the conserved W972 to an alanine and an arginine. Minimal differences were observed in the solution structures of WT and the W972A mutant, whereas the W972R mutant distorts the last turn of the α-helix, introducing an additional positive surface charge due to the introduction of a bulky, positively charged side chain [82]. The W972R mutant lengthened the distance between the zinc ions compared to the W972A mutant, where the distance is shortened. Relaxation studies ultimately revealed that the W972R mutant exhibits higher mobility in the C-terminal end in the α-helix compared to WT and W972A. Additionally, in vivo luciferase reporter assays showed that the W972R mutation does not retain its ligase activity, suggesting that this mutant is inactive [82]. What remains to be explored is how small structural changes within the RING core affect the formation of the E2:E3 complex.

**Figure 6:**
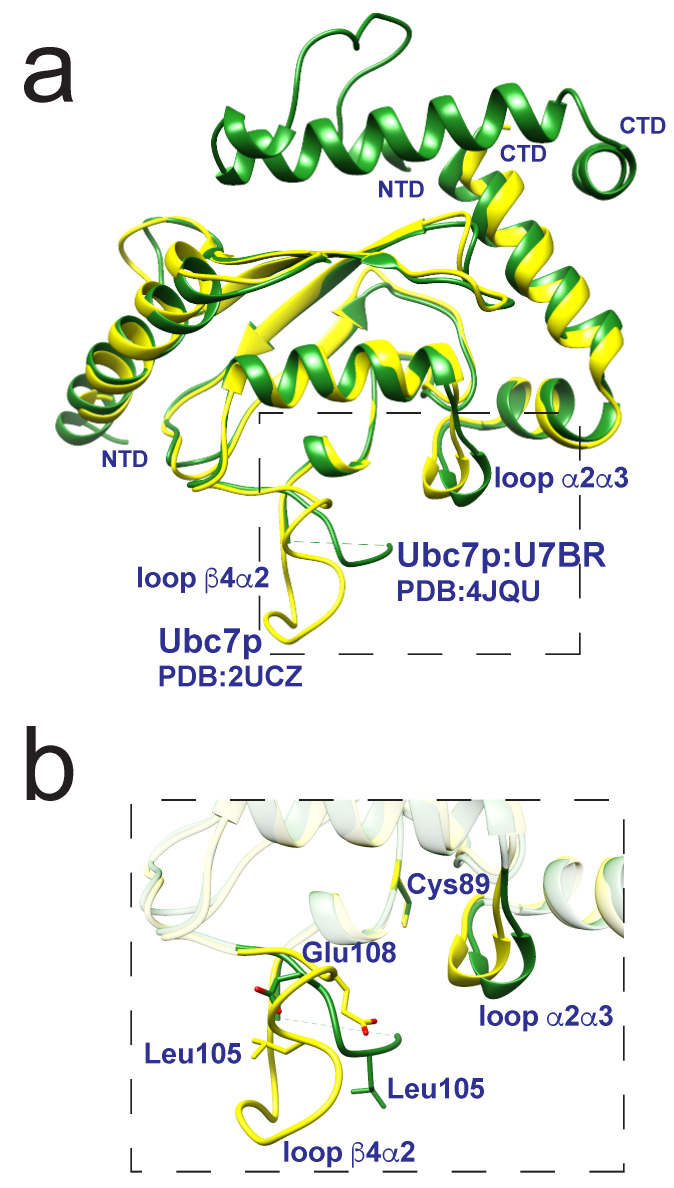
Structural representations (**a**) of Ubc7p (yellow, PDB 2UCZ) overlaid with Ubc7p:U7BR (green, PDB 4JQU) highlighting the minimal differences within the two structures with a dashed box. A zoomed-in representation (**b**) showing loop α${}_{2}$α${}_{3}$ shifts away from the Cys89 once U7BR binds to Ubc7p. This interaction also introduces changes in loop β${}_{4}$α${}_{2}$, highlighted by the structural differences in side-chains Leu105 and Glu108. Figure adapted from Metzger et al. [84].

One of the complexities of the E2:E3 pairings and the establishment of combinatorial regulation involves the roles of secondary binding regions or noncanonical binding partners [49,83,84,85,86,87]. The principles involved and the role of dynamics have been explored in two analogous ERAD E2:E3 pairs, which reveal the allosteric effects of backside binding domains on the recognition and affinity of binding the E3 RING domain. First, in yeast, the membrane-associated protein Cue1p recognizes the E2 Ubc7p via a domain denoted U7BR (Cue1p${}_{U7BR}$). Binding of the U7BR to Ubc7p enhances ubiquitin transfer from Ubc7p stimulated by RING-dependent and -independent mechanisms [84]. Crystal structures revealed disordered portions of the β${}_{4}$α${}_{2}$ loop in Ubc7p upon backside binding of U7BR [84]. The β${}_{4}$α${}_{2}$ loop in U7BR-bound Ubc7p was found to have elevated mobility, based on lower hetNOE values compared to the apo structure, which induced the α${}_{2}$α${}_{3}$ loop to shift away from the catalytic cysteine (Figure 6) [84]. The backside binding of U7BR of Cue1p to Ubc7p induces an allosteric effect in Ubc7p leading to an increased affinity of RING domain, supported through CSP measurements and RING-dependent ubiquitin discharge assays. Ubc7p exhibits multiple in vivo ubiquitination activities, for example, Ubc7p (i) acts only as a ubiquitin chain elongator with the E3 Doa10 yet (ii) acts as both a primer (directly attaching ubiquitin to substrate) and a chain elongator with the E3 Hrd1 [15]. It is not yet clear how U7BR modulates the dynamics within Ubc7p.

**Figure 7:**
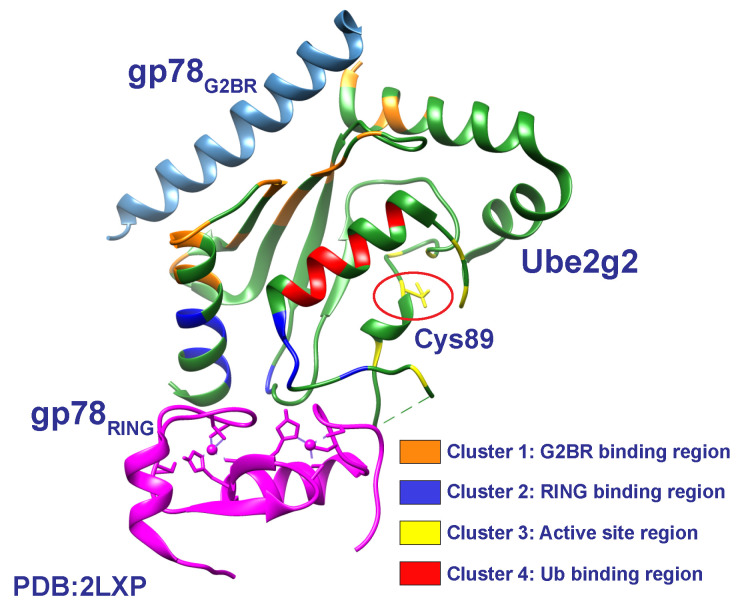
Structural representation of the ternary complex, Ube2g2:G2BR:RING. NMR relaxation experiments identified four clusters of residues that increased their rigidity going from free Ube2g2 to G2BR-bound Ube2g2. The four clusters include the backside binding region that binds G2BR (orange), the RING binding domain (blue), the active-site cysteine and surrounding loops (β${}_{4}$α${}_{2}$ and α${}_{2}$α${}_{3}$) (yellow), and the residues that interact with the conjugate ubiquitin (red). All four clusters are shown to interact with gp78 domains. Figure adapted from Chakrabarti et al. [23].

Second, in humans, the E3 gp78 binds to its cognate E2, Ube2g2, through two domains: the G2BR domain (gp78${}_{G2BR}$), which becomes ordered upon binding Ube2g2; and the RING domain (gp78${}_{RING}$) [49,83,88]. The binary G2BR:Ube2g2 complex forms with nanomolar affinity and exhibits slow exchange by CSP measurements [83,84]. The binding of G2BR to Ube2g2 induces allosteric effects, significantly increasing the affinity for RING domain [49,83]. Crystal structures revealed that the flexibility seen in α${}_{2}$α${}_{3}$ and β${}_{4}$α${}_{2}$ loops of Ube2g2 become stabilized upon G2BR binding, and these loops become dynamic upon RING binding in the formation of the G2BR:Ube2g2:RING ternary complex [49]. The critical structural feature of the ternary complex is the salt bridge formation between Ube2g2${}_{E108}$ and RING${}_{R379}$ [49]. Mutations introduced to disrupt the salt bridge within the ternary complex portrayed a dramatically decreased rate of autoubiquitination [49]. Using hetNOE and residual dipolar coupling (RDCs) experiments to explore ps-to-ms timescale dynamics (Figure 2), the majority of Ube2g2 residues, with the exception of four clusters, were found to be relatively rigid in the free form and remained rigid in the G2BR-bound form. The four clusters include the backside region that binds G2BR, the RING binding region, the active-site cysteine and surrounding loops (β${}_{4}$α${}_{2}$ and α${}_{2}$α${}_{3}$), and the residues that interact with the conjugate ubiquitin (Figure 7) [23]. To explore the μs-to-ms timescale dynamics, experiments were performed using ^15^N CPMG-RD on a Ube2g2${}_{C89K}$ variant, where dynamics were indistinguishable between WT Ube2g2. Dispersion was undetectable at room temperature, indicating that any conformational dynamics may be outside the timescale detectable by CPMG (Figure 2) [23]. In order to confirm and visualize these dynamics, CPMG experiments were performed at 1.5 °C. Free Ube2g2 was used as a proxy for the population redistribution of Ube2g2∼Ub upon binding of G2BR and RING domains. Relaxation dispersion data for Ube2g2 alone found 59 residues distributed among the previously defined four clusters of Ube2g2 that displayed motion on the μs timescale [23]. With the addition of G2BR to form the binary complex, conformational dynamics become quenched as all four clusters experience a loss of motion and populate a single state. Upon formation of the ternary complex with RING, known to trigger the transfer of ubiquitin, a reemergence of conformational dynamics is observed by reintroducing motion in all four clusters of Ube2g2 and a different energy landscape of conformations [23]. The NMR dynamics data combined with MD trajectories suggest a dynamic energy landscape consisting of multiple conformations, whose populations shift in concert with recognition of and response to binding domains. For example, the conformation of Ube2g2 favorable to salt-bridge formation with RING is a minor state of the E2, which correlates with weak affinity for RING. Upon binding of G2BR to the backside of the E2, the dynamic population of this minor state is increased, thus lowering the energy barrier to salt-bridge formation and increasing the affinity for RING [23]. Finally, upon binding of RING, the landscape shifts to a dynamic distribution of multiple states poised for ubiquitin transfer.

Since the binding surfaces of E3 RING domains that bind to E2s are broadly similar across the family, the recognition of the E2-specific backside binding regions, e.g., gp78${}_{G2BR}$ and Cue1p${}_{U7BR}$, appear to dynamically shift the population of the E2 conformation to a favorable state and increase the affinity [83]. Hence, there may be a recognition network operative for E2:E3 pairs that involves dynamics and allosteric shifting of conformational populations to regulate ubiquitination. Clearly, further work is required to examine such hypotheses, and the detection of these motions at faster timescales and for larger complexes is quite a challenge. Fortunately, new tools (HARD and E-CPMG) are being developed in order to further investigate the broader impact of these motions [26,89]. It remains to be determined if similar, yet distinct, energy landscapes may exist for other E2:E3 pairs. The answers may provide what role dynamics play in regulation within ubiquitination pathway.

## 5. Conclusions

For many years, researchers have studied the individual components of the ubiquitination pathway providing a foundational understanding of the proteins, complexes, and, to a lesser extent, the intrinsic motions of these proteins. The combinatorial complexity introduced by the formation of larger complexes and the experimental challenges that arise highlight the need for new experimental techniques to explore molecular recognition events and their catalytic mechanisms. The works summarized in this review clearly show that activation of E2s by RING-type E3s proceed by an allosteric mechanism, in which RING and other potential binding domains alter the conformational dynamics to favor more catalytically competent states. The current model of the active conformation consists of the hydrophobic patch of ubiquitin interacting with helix α${}_{2}$ of E2 stabilizing the closed conformation, and the substrate lysine (or lysine on Ub from a ubiquitinated substrate) is poised for nucleophilic attack. Additionally, a conserved asparagine in E2s is responsible for the stabilization of the oxyanion in the transition state for E2:RING E3 complexes [9], yet it does not perform this role in HECT-type E3 ubiquitin transfer. The motions within the 10–100 μs timescale are inaccessible to previous experimental techniques (Figure 2) [24], leading to an issue of quantitation and relation of the rates of these conformational dynamics to known biochemical parameters.

Given our current model of understanding, there are many questions that are left unanswered. For instance, are other E3 domains involved in ubiquitin transfer, how are these other E3 domains involved, are there other binding partners additional to E2s and E3s, how are substrates recognized by E2:E3 complexes, how is processivity achieved, and how are different architectures of ubiquitin chains are formed? New geoHARD NMR methods, developed in our laboratory, allow us to access the timescales previously restricted by currently available techniques [25,26,48] and apply them to larger molecular species. These methods should be combined with CEST or ZZ-exchange [42,90] to reveal the full energy and dynamics landscape from the μs-to-ms time regime (Figure 2). With these new tools in hand, as well as methods currently being developed in other laboratories, the opportunity exists to explore conformational dynamics of these larger complexes and begin to answer some of these questions.

## Abbreviations

The following abbreviations are used in this manuscript:

Ub	ubiquitin
E1	ubiquitin-activating enzyme
E2	ubiquitin-conjugating enzyme
E3	ubiquitin-ligating enzyme
E2∼Ub	thioester-linked E2∼Ub conjugate
E2-Ub	nonthioester-linked E2-Ub conjugate
HECT	homologous to E6AP C-terminal
RING	Really Interesting New Gene
NMR	nuclear magnetic resonance
R${}_{1}$	longitudinal relaxation rate
R${}_{2}$	transverse relaxation rate
R${}_{1\rho }$	rotating frame longitudinal relaxation rate
hetNOE	heteronuclear Overhauser effect
S^2^	rigidity of the amide bond vector
τ${}_{e}$	intramolecular tumbling time
τ${}_{c}$	overall molecular tumbling time
CPMG-RD	Carr–Purcell–Meiboom–Gill relaxation dispersion
HARD	heteronuclear adiabatic relaxation dispersion
UEV	ubiquitin E2 variants
CSP	chemical shift perturbation
FRET	Förster Resonance Energy Transfer
ERAD	endoplasmic reticulum associated degradation
PRE	paramagnetic relaxation enhancement
RDC	residual dipolar coupling
CEST	chemical exchange saturation transfer
MD	molecular dynamics
SAXS	small-angle x-ray scattering
